# Tools for delivering entomopathogenic fungi to malaria mosquitoes: effects of delivery surfaces on fungal efficacy and persistence

**DOI:** 10.1186/1475-2875-9-246

**Published:** 2010-08-27

**Authors:** Ladslaus L Mnyone, Matthew J Kirby, Dickson W Lwetoijera, Monica W Mpingwa, Emmanuel T Simfukwe, Bart GJ Knols, Willem Takken, Tanya L Russell

**Affiliations:** 1 Biomedical and Environmental Group, Ifakara Health Institute, P.O. Box 53, Off Mlabani Passage, Ifakara, Tanzania; 2 Laboratory of Entomology, Wageningen University & Research Centre, P.O. Box 8031, 6700 EH, Wageningen, The Netherlands; 3 Vector Group, Liverpool School of Tropical Medicine, Liverpool, L3 5QA, UK; 4 Pest Management Centre, Sokoine University of Agriculture, P.O. Box 3110, Morogoro, Tanzania; 5 Department of Zoology and Marine Biology, University of Dar es Salaam, P.O. Box 35064, Dar es Salaam, Tanzania; 6 Division of Infectious Diseases, Tropical Medicine & AIDS Academic Medical Center, F4-217, Meibergdreef 9, 1105 AZ, Amsterdam, The Netherlands; 7 The University of Queensland, School of Population Health, Australian Centre for Tropical and International Health, Brisbane, 4006, Australia

## Abstract

**Background:**

Entomopathogenic fungi infection on malaria vectors increases daily mortality rates and thus represents a control measure that could be used in integrated programmes alongside insecticide-treated bed nets (ITNs) and indoor residual spraying (IRS). Before entomopathogenic fungi can be integrated into control programmes, an effective delivery system must be developed.

**Methods:**

The efficacy of *Metarhizium anisopliae *ICIPE-30 and *Beauveria bassiana *I93-825 (IMI 391510) (2 × 10^10 ^conidia m^-2^) applied on mud panels (simulating walls of traditional Tanzanian houses), black cotton cloth and polyester netting was evaluated against adult *Anopheles gambiae *sensu stricto. Mosquitoes were exposed to the treated surfaces 2, 14 and 28 d after conidia were applied. Survival of mosquitoes was monitored daily.

**Results:**

All fungal treatments caused a significantly increased mortality in the exposed mosquitoes, descending with time since fungal application. Mosquitoes exposed to *M. anisopliae *conidia on mud panels had a greater daily risk of dying compared to those exposed to conidia on either netting or cotton cloth (*p *< 0.001). Mosquitoes exposed to *B. bassiana *conidia on mud panels or cotton cloth had similar daily risk of death (*p *= 0.14), and a higher risk than those exposed to treated polyester netting (*p *< 0.001). Residual activity of fungi declined over time; however, conidia remained pathogenic at 28 d post application, and were able to infect and kill 73 - 82% of mosquitoes within 14 d.

**Conclusion:**

Both fungal isolates reduced mosquito survival on immediate exposure and up to 28 d after application. Conidia were more effective when applied on mud panels and cotton cloth compared with polyester netting. Cotton cloth and mud, therefore, represent potential substrates for delivering fungi to mosquitoes in the field.

## Background

To eliminate malaria, vector control programmes will need to incorporate novel tools to complement the use of insecticide-treated bed nets (ITNs) and indoor residual spraying (IRS). Both ITNs and IRS are highly effective against anthropophagic and endophilic species, but their efficacy is threatened by emergence of resistance to synthetic insecticides [1,2]. Therefore, the growing demand of the global community for non-chemical control tools has refocused research objectives to address the practical aspects of biological control tools that have previously had limited uptake. Biological control tools have several advantages over chemical-insecticides. The most important ones include reduced risk of host resistance and minimal risk to the environment and living organisms [3,4]. Currently, a number of novel tools based on biological interactions are undergoing development including fungal, bacterial, viral and protozoan pathogens [5]. Of these, entomopathogenic fungi show considerable promise for development as biopesticides [6-10]. Fungus production and application all involve relatively simple infrastructure and processes [4,10], which can be readily adopted in malaria-endemic countries. Although fungal infection reduces the fecundity of female mosquitoes, they are still able to pass their genes to the subsequent generations, thus weakening the selection for resistance development [3,11,12]. Fungal infection does not cause instant mortalities to mosquitoes as it is to the chemical insecticides [12].

Before entomopathogenic fungi can be integrated into control programmes, additional information regarding isolate selection, optimisation of production and formulation is required. While many successful laboratory evaluations of the efficacy of entomopathogenic fungi have been conducted [12,13], more research evaluating various formulations, delivery techniques and formats remains essential. Fungal formulations could be used to target either host-seeking and/or resting mosquitoes. Host-seeking mosquitoes could be targeted when entering a house through the eaves [14,15], or when attacking a host under a bed net [16]. Resting mosquitoes could be targeted indoors on walls [7,17] or both indoors and outdoors using a point source decoy resting site, e.g. resting boxes, clay pots, or black cotton cloth attached to the roof or walls [6,7]

The aim of the current study was to test different surface substrates that can be used to infect mosquitoes with fungal conidia and to examine whether the surface affected the availability, efficacy and persistence of the conidia.

## Methods

### Mosquito rearing and maintenance

*Anopheles gambiae sensu stricto *were reared at the Ifakara Health Institute (IHI) insectary (colony established in 1996, Njage village, Tanzania). This colony is being supplemented with newly collected field mosquitoes to maintain its vigor. Larval and adult stages of the mosquitoes were raised using methods described by Huho et al [18]. All bioassays were conducted on 3-6 d old adult female mosquitoes that had had access to 9% glucose/water (w/v) since emergence. During experiments, mosquitoes were maintained on 9% glucose/water (w/v) solution.

### Fungal isolates and formulation

Two fungal isolates, *Metarhizium anisopliae *var. *anisopliae *Sorokin isolate ICIPE-30 and *Beauveria bassiana *Vuillemin isolate I93-825 (IMI 391510) were used in all bioassays. *Metarhizium anisopliae *was isolated from the maize stalk borer, *Busseola fusca *(Lepidoptera, Noctuidae), in 1989 in Western Kenya and imported as dry conidia from Wageningen University, The Netherlands (courtesy F. van Breukelen, Wageningen University). *Beauveria bassiana *was isolated from a chrysomelid beetle (Coleoptera) in the USA and imported as dry conidia from the Commonwealth Scientific and Industrial Research Organisation (CSIRO), Australia and Penn State University, USA (courtesy M. Thomas & N. Jenkins, Penn State University). Dry conidia of *M. anisopliae *were produced at IHI, after passaging and re-isolation from infected mosquito cadavers. Conidia were harvested from 15 day old cultures grown on rice grains. The viability of conidia (>85% germination) was confirmed before each bioassay by inoculating 0.2 ml of suspended 10^7 ^conidia/ml onto SDA. Three agar plates were used per each fungal isolate. After 16 - 18 h incubation at 26°C and 80% Relative humidity (RH) the germination of 300 conidia in total for each of the three agar plates was quantified from different fields of the inoculum. The conidia viability was then calculated as an average of the percentage germination in the three agar plates.

Conidia were formulated in oil before application. Fungal stock solution was prepared by suspending 1-2 g of conidia in 20 ml of a 1:1 mixture of highly refined Enerpar (Enerpar M002^®^, BP South Africa LTD) or Shellsol (Shellsol T^®^, South Africa Chemicals) oils. The Enerpar and Shellsol oils have no effect on conidia germination or infectivity (L.M., unpublished data). To homogenize the mixture it was shaken vigorously, vortexed for 25 sec and then sonicated for 3 min in an ultrasonic bath, 40 kHz frequency (Langford Electronics, UK). Dilutions of 1:10, 1:100 and 1:1000 were then prepared in the same oil mixture and the concentration of conidia calculated using a Neubauer haemocytometer (Hausser Scientific Horsham PM, USA) and compound microscope (Leica ATC™ 2000, Buffalo, NY 14240, U.S.A) at 400 × magnification. The solution was adjusted to 2 × 10^10 ^conidia m^-2 ^by further dilution in oil.

### Delivery substrates

**Figure 1:**
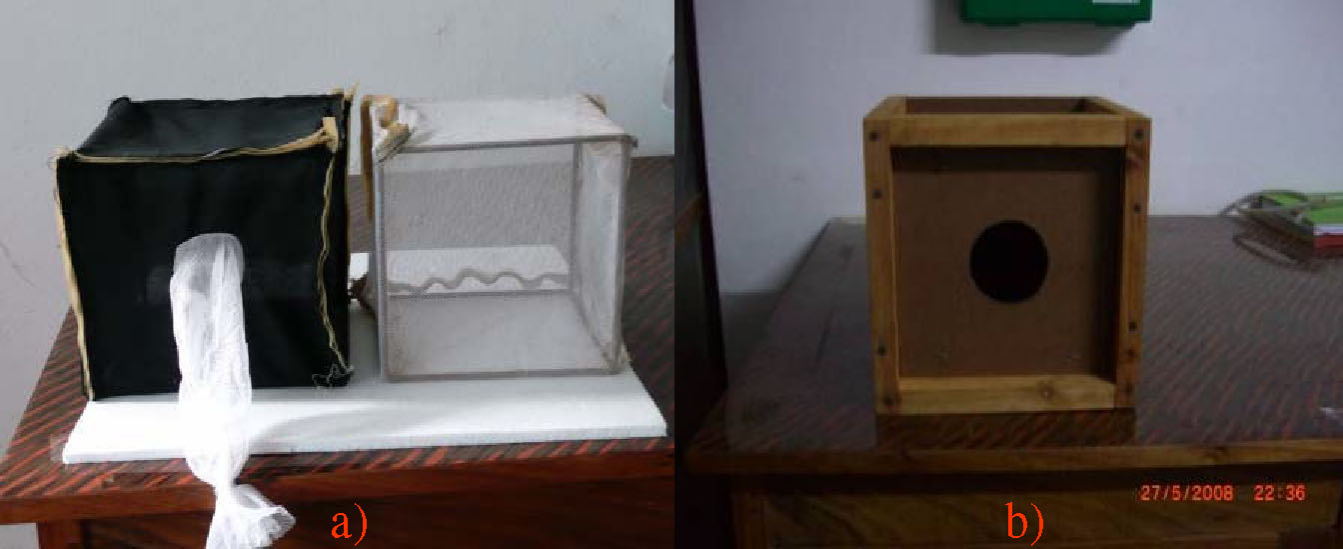
**A photograph of a) black cloth and netting cages, b) cage made with mud-lined panels**.

The delivery surfaces examined were:

1) polyester netting (intended to target host-seeking mosquitoes) was obtained from Safi Net™, A to Z Textile Mills Ltd, Arusha, Tanzania. White netting was used.

2) mud panels similar to the walls of adobe houses (intended to target resting mosquitoes). About 20 mm layer of mud, collected from natural soils near Ifakara, was lined onto the inner surface of plywood panels. The panels were kept to dry under room temperature for about three weeks. Panels had screws, thus the individual sections could be assembled to obtain mud cages for the bioassays (Figure 1).

3) black cotton cloth (intended to target resting mosquitoes). The cloth was obtained from the local shops, Ifakara, Tanzania.

The fungal solution was applied to the delivery surfaces using a hand-held air compressor sprayer (Minijet^®^, SATA, Germany) held 50 cm away and at a right angle to the surface. About 23 ml of fungal formulation (2 × 10^10 ^conidia m^-2^) was applied onto each of the surfaces. Treated materials were left to dry for 48 h. Sections of netting and black cotton cloth were then joined using Velcro strips, to fit over 20 cm^3 ^wire frame cages. Mud-lined plywood panels were assembled into 20 × 20 × 20 cm cages. Mosquitoes were then exposed to the treated surfaces (Figure 1) as described in the bioassay procedures below.

### Bioassay procedures

To test the efficacy of conidia applied to different delivery substrates, 40-55 adult *An. gambiae *s.s mosquitoes were introduced to four replicate conidia-treated and oil treated control cages for 6 h. After exposure, mosquitoes were transferred to separate untreated cages (9 × 9 × 9 cm) and maintained at 26 ± 1°C and 90 ± 5% relative humidity (RH) with access to 9% glucose/water (w/v) solution *ad libitum*. Mosquito survival was monitored daily for a maximum of 28 d. Dead mosquitoes were collected and put onto moist filter paper in petri dishes, sealed with parafilm, and kept at 26 ± 1°C and 90 ± 5% RH for 3-4 d, after which they were examined for evidence of fungal sporulation. After the initial evaluation, all treated surfaces were stored at 26 ± 1°C and 90 ± 5% RH and separate mosquito cohorts were exposed 14 and 28 d later to determine the persistence of conidia on different delivery surfaces.

### Data analysis

Mosquito survival data were analysed using Cox regression to determine the relative risk of dying (hazard ratios) for the specific treatment group compared with the control group and with each other. The explanatory factors included in the analysis were treatment, delivery surface and time of exposure post-application. The Kaplan-Meier method was applied to obtain median survival times for treated and untreated groups of mosquitoes. Survival curves were considered not statistically different at *p *> 0.05. Bonferroni method was employed to compensate for multiple comparisons. SPSS version 16 (SPSS Inc., Chicago, IL) was applied.

## Results

Overall, fungal infection reduced the median survival time (MST) of fungal-exposed mosquitoes compared to the controls regardless of delivery surface or time of exposure post-application (Table 1). The daily risk of dying for mosquitoes exposed to either *M. anisopliae *(Hazard Ratio [HR] = 2.72 [95% CI = 2.58 - 2.86], *p *< 0.001) or *B. bassiana *(HR = 2.23 [95% CI = 2.12 - 2.34], *p *< 0.001) was more than two-fold higher than that of control mosquitoes (Table 2). For *M. anisopliae*, the risk of death for mosquitoes exposed to conidia on mud panels was higher than that for mosquitoes exposed to conidia on either polyester netting (HR = 1.17 [95% CI = 1.1 - 1.24], *p *< 0.001) or cotton cloth (HR = 1.11 [95% CI = 1.05 - 1.18], *p *< 0.001) regardless of the time at which mosquitoes were exposed post conidia application (Table 2). Mosquitoes exposed to *B. bassiana *on mud panels had a similar risk of death to mosquitoes exposed to this fungus on cotton cloth (HR = 0.96 [95% CI = 0.91 - 1.01], *p *= 0.14) regardless of the time at which mosquitoes were exposed post conidia application. The risk of death for mosquitoes exposed to conidia on the mud panels and cotton cloth was higher than that for mosquitoes exposed to conidia on the polyester netting (mud panel: HR = 1.17 [95% CI = 1.11 - 1.24]; cotton cloth: HR = 1.19 [95% CI = 1.14 - 1.23], *p *< 0.001, Table 2). As such, in overall, the poorest performance was consistently recorded for the polyester netting material.

**Table 1:** Median survival times (MST ± SE) of *Anopheles gambiae s.s*. exposed to *Metarhizium anisopliae *ICIPE-30 and *Beauveria bassian a *I93-825 at day 2, 14 and 28 post-application of fungus to mud panel, polyester netting and black cotton cloth

Fungus isolate	Delivery surfaces	Days post application	MST ± SE		**χ**^**2 **^**value**	*p *value
			**Treatment**	**Control**		
***M. anisopliae***	Mud panel	2	6 ± 0.62	13 ± 0.68	125.64	<0.001
		14	7 ± 0.30	16 ± 0.94	70.37	<0.001
		28	9 ± 0.02	14 ± 0.72	62.80	<0.001
	
	Polyester netting	2	6 ± 0.40	14 ± 0.85	108.04	<0.001
		14	11 ± 0.51	16 ± 1.01	58.04	<0.001
		28	11 ± 0.82	15 ± 0.70	62.89	<0.001
	
	Cotton cloth	2	5 ± 0.49	14 ± 0.61	61.81	<0.001
		14	9 ± 0.37	17 ± 0.70	80.57	<0.001
		28	11 ± 0.38	15 ± 0.72	50.46	<0.001

***B. bassiana***	Mud panel	2	8 ± 0.27	13 ± 0.68	86.09	<0.001
		14	9 ± 0.45	16 ± 0.94	62.52	<0.001
		28	10 ± 0.47	14 ± 0.72	41.08	<0.001
	
	Polyester netting	2	10 ± 0.62	14 ± 0.61	54.90	<0.001
		14	11 ± 0.62	17 ± 0.70	33.01	<0.001
		28	12 ± 0.66	15 ± 0.72	27.88	<0.001
	
	Cotton cloth	2	5 ± 0.24	14 ± 0.85	156.47	<0.001
		14	10 ± 0.50	16 ± 1.01	76.42	<0.001
		28	10 ± 0.44	15 ± 0.70	52.64	<0.001

**Table 2:** Mortality hazard ratios of mosquitoes exposed to *Metarhizium anisopliae *ICIPE-30 and *B. bassian a *I93-825 at day 2, 14 and 28 post-application of fungus to mud panel, polyester netting and cotton cloth

Factor	Comparisons	HR	95% C.I	P value
***Metarhizium anisopliae *ICIPE-30**				
**Treatment**	Treatment vs control	2.72	2.58 - 2.86	<0.001

**Surfaces**	Mud panel vs polyester netting	1.17	1.1 - 1.24	<0.001
	Mud panel vs cotton cloth	1.12	1.05 - 1.18	<0.001
	Cotton cloth vs polyester netting	0.96	0.91 - 1.02	0.17

**Days post-application**	2 vs 14	0.95	0.9 - 1	0.05
	2 vs 28	1.12	1.06 - 1.19	<0.001
	14 vs 28	1.05	1 - 1.11	0.11

***Beauveria bassiana *I93-825**				
**Treatment**	Treatment vs control	2.23	2.12 - 2.34	<0.001

**Surfaces**	Mud panel vs polyester netting	1.17	1.11 - 1.24	<0.001
	Mud panel vs cotton cloth	0.96	0.91 - 1.01	0.14
	Cotton cloth vs polyester netting	1.19	1.14 - 1.23	<0.001

**Days post-application**	2 vs 14	1.13	1.03 - 1.25	0.01
	2 vs 28	1.17	1.06 - 1.28	0.002
	14 vs 28	0.99	0.93 - 1.06	0.82

**Figure 2:**
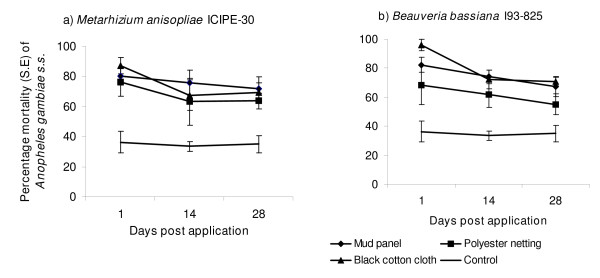
**Percentage mortality (± SE) of *An. gambiae s.s*. mosquitoes 12 d post exposure to *M. anisopliae *ICIPE-30 and *B. bassiana *I93-825 applied on mud panel, polyester netting and black cotton cloth, at 2, 14 and 28 d post treatment**.

The effect of conidia on mosquito survival generally declined over time post-application for all delivery surfaces examined (Figure 2). Mosquitoes exposed to *M. anisopliae *2 d and 14 d post-application, had a similar risk of death (HR = 0.95 [95% CI = 0.9 - 1], *p *= 0.05). The residual efficacy of conidia of *M. anisopliae *had significantly declined by 28 d post-application, with the relative risk of death for mosquitoes exposed at 2 d post-application being higher (HR = 1.12 [95% CI = 1.06 - 1.19], *p *< 0.001, Table 2). The residual efficacy of *B. bassiana *declined a little more sharply with mosquitoes exposed at 2 d post-application having a higher risk of death than mosquitoes exposed both 14 d (HR = 1.17 [95% CI = 1.03 - 1.25], *p *= 0.01) and 28 d (HR = 1.17 [95% CI = 1.06 - 1.28], *p *= 0.002) post application. Mosquitoes that were exposed to *B. bassiana *at 14 d and 28 d post-application had similar risk of death (HR = 0.99 [95% CI = 0.93 - 1.06], *p *= 0.82, Table 2). Nonetheless, 28 d post-application conidia of both fungi still infected and killed 73 - 82% of mosquitoes by day 14.

## Discussion

This study was intended to provide fundamental information for developing delivery formats in advance of field evaluations of entomopathogenic fungi against malaria vectors. Consistent with published literature [6,12,13], this study indicated that infection with either *M. anisopliae *or *B. bassiana *significantly reduced the survival of female *An. gambiae s.s*. mosquitoes. The efficacy of fungal formulations (measured by reduction of adult survival) varied among the candidate delivery materials. The efficacy of *M. anisopliae *conidia was highest when applied on mud panels compared with cotton cloth or polyester netting. For *B. bassiana*, the efficacy of conidia was highest when applied to either mud panels or cotton cloth compared with polyester netting. The lowest efficacy of either fungal isolate was consistently recorded for the polyester netting. Rapid decline in conidial efficacy over time after application on the netting has also been reported in other studies [19]. Variations in efficacy of treatment between surfaces of different material are not unique; previous research into pyrethroid insecticides for impregnating bed nets revealed that efficacy is dependent on the type of fabric used, with polyester being more effective than nylon and cotton [20]. In the present study, polyester netting may have reduced the efficacy of both fungal species, possibly through poor conidia attachment due to its smooth fibres and/or chemical effects from the netting itself or chemicals used to soften polyester fibres.

Even though the residual activity of fungal isolates declined over time, conidia remained pathogenic up to 28 d post application (and possibly longer) and were still able to infect and kill 73 - 82% of mosquitoes within 14 d. When conidia were applied on the netting, the residual activity declined much more rapidly compared with mud panels and cotton cloth. Decline in the residual activity of conidia has also been reported elsewhere [6,7]. The residual activity of fungal conidia appears to decline at comparable rates to other biopesticides, for example *Bacillus thuringiensis *[21,22]. The residual activity of chemical insecticides also declines with time, but compared to biopesticides their persistence is often longer [23], which is partially dependent on dose. Fungal formulation that can infect and kill at least 50% of the host-seeking mosquitoes for over two months after field application is desirable, as would exert considerable epidemiological impact on malaria transmissions [7,16].

It is important to note that the treatable surface area of polyester netting was much smaller than mud and cotton cloth due to the holes in this material (25 holes/cm^2^). As such the netting had a smaller surface area for both conidial attachment and exposure of mosquitoes. The higher efficacy of fungus observed when applied to mud and cotton cloth could therefore have simply been due to availability of more conidia per unit area, maximizing mosquito exposure and probability of picking up more conidia.

The pathogenic effect of fungus declined over time in different substrates, with efficacy declining the fastest on the polyester netting. In the initial exposure (2 d post application), many conidia could have been readily available on the surface of each substrate. With time, however, conidial viability and virulence could have decreased thus the conidia became less efficacious to mosquitoes. This may have accounted for a quick drop in efficacy of fungi applied on cotton cloth and netting between the initial and subsequent exposures. A drop in efficacy could also be explained by reduced virulence of the conidia. For the mud panel, the decline was fairly constant. Others have reported a decline in residual activity resulting from fluctuating environmental conditions [7], but this was unlikely the cause here since experiments were done under stable and controlled conditions.

The aim was to develop a delivery tool(s) that facilitates rapid dissemination of fungal conidia to mosquitoes and remains effective for a prolonged amount of time. The high mortality (82% within 14 d) since exposure of mosquitoes to conidia on mud and cotton cloth 28 d post application emphasizes the potential of these two surfaces as target tools. The delivery surfaces examined in the current study were selected because they could be easily adapted for practical dissemination of conidia under realistic field conditions. Cotton cloth could be placed to partially cover eave openings, on ceilings [7], and on internal surface of resting traps (e.g. lure and kill with resting stations) [24]. For mud panels, conidia could be applied using indoor residual spraying. In an effort to also target outdoor resting mosquitoes, odour-baited traps [25] made from mud panels could be useful. Results of this study support further research into any of these suggestions that may effectively disseminate mosquito-killing conidia while being practical for end users of the technology.

## Conclusions and recommendations

Mosquitoes exposed to entomopathogenic fungi expressed a reduced survival from conidia used between two and 28 days after application. Conidia were more effective when applied on mud panels and cotton cloth compared with polyester netting. Cotton cloth and mud, therefore, present useful and practical tools for applying fungi against resting mosquitoes in the field. These tools should be used such that mosquito contact to conidia is maximized in order to correctly predict the efficacy and residual activity of fungi.

## Competing interests

The authors declare that they have no competing interests.

## Authors' contributions

Conceived and designed the experiments: LLM TLR BJK WT. Performed the experiments: LLM DWL MWM. Analyzed the data: LLM TLR MJK. Wrote the paper: LLM TLR MJK. Reviewed the paper: BJK WT. All authors read and approved the final manuscript.

## References

[B1] N'Guessan R Corbel V Akogbéto M Rowland M Reduced efficacy of insecticide-treated nets and indoor residual spraying for malaria control in pyrethroid resistance area, Benin Emerg Infect Dis 2007 13 199 206 10.3201/eid1302.060631 17479880 PMC2725864

[B2] Moreno M Vicente JL Cano J Berzosa PJ de Lucio A Nzambo S Bobuakasi L Buatiche JN Ondo M Micha F Knockdown resistance mutations (kdr) and insecticide susceptibility to DDT and pyrethroids in *Anopheles gambiae *from Equatorial Guinea Trop Med Int Health 2008 13 430 3 10.1111/j.1365-3156.2008.02010.x 18397404

[B3] Ffrench-Constant RH Something old, something transgenic, or something fungal for mosquito control? Trends Ecol Evol 2005 20 577 579 10.1016/j.tree.2005.08.007 16701437

[B4] Strasser H Vey A Butt TM Are there any risks in using entomopathogenic fungi for pest control, with particular reference to the bioactive metabolites of *Metarhizium*, *Tolypocladium *and *Beauveria *species Biocontrol Sci Techn 2000 10 717 735 10.1080/09583150020011690

[B5] Read AF Thomas MB Microbiology: Mosquitoes Cut Short Science 2009 323 5910 51 2 10.1126/science.1168659 19119208

[B6] Farenhorst M Farina D Scholte EJ Takken W Hunt RH Coetzee M Knols BGJ African water storage pots for the delivery of the entomopathogenic fungus *Metarhizium anisopliae *to the malaria vectors *Anopheles gambiae s.s*. and *Anopheles funestus* Am J Trop Med Hyg 2008 78 910 916 18541768

[B7] Scholte EJ N'gabi K Kihonda J Takken W Paaijmans K Abdulla S Killeen GF Knols BGJ An entomopathogenic fungus for control of adult malaria mosquitoes Science 2005 308 1641 1642 10.1126/science.1108639 15947190

[B8] Luz C Fargues J Romana CA Moreno J Goujet R Rougier M Grunewald J Potential of entomopathogenic hyphomycetes for the control of the triatomine vectors of Chagas' disease Proceedings of VIth International Coll Invertebrate Pathology Microbiological Control 1994 1 272 276

[B9] Samuels RI Coracini DLA Selection of *Beauveria bassiana *and *Metarhizium anisopliae *isolates for the control of *Blissus antillus *(Hemiptera: Lygaeidae) Sci Agric 2004 61 271 275 10.1590/S0103-90162004000300005

[B10] Zimmermann G The entomopathogenic fungus *Metarhizium anisopliae *and its potential bio-control agent Pest Sci 1993 37 375 379 10.1002/ps.2780370410

[B11] Read AF Lynch PA Thomas MB How to make evolution-proof insecticides for malaria control PLoS Biol 2009 7 e1000058 10.1371/journal.pbio.1000058 19355786 PMC3279047

[B12] Blanford S Chan BHK Jenkins N Sim D Turner RJ Read AF Thomas MB Fungal pathogen reduces potential for malaria transmission Science 2005 308 1638 1641 10.1126/science.1108423 15947189

[B13] Achonduh OA Tondje PR First report of pathogenicity of *Beauveria bassiana *RBL1034 to the malaria vector, *Anopheles gambiae *s.l (Diptera; Culicidae) in Cameroon Afr J Biotechnol 2008 7 931 935

[B14] Njie M Dilger E Lindsay SW Kirby MJ Importance of eaves to house entry by anopheline, but not culicine, mosquitoes J Med Entomol 2009 46 505 510 10.1603/033.046.0314 19496420

[B15] Lines JD Myamba J Curtis CF Experimental hut trials of permethrin-impregnated mosquito nets and eave curtains against malaria vectors in Tanzania Med Vet Entomol 1987 1 37 51 10.1111/j.1365-2915.1987.tb00321.x 2979519

[B16] Hancock PA Combining fungal biopesticides and insecticide-treated bednets to enhance malaria control PloS Comput Biol 2009 5 e1000525 10.1371/journal.pcbi.1000525 19798436 PMC2742557

[B17] Gillies MT Studies in house leaving and outside resting of *Anopheles gambiae *Giles and *Anopheles funestus *Giles in East Africa. II. The exodus from houses and the house resting population Bull Entomol Res 1954 45 375 387 10.1017/S000748530002719X

[B18] Huho BJ Ng'habi KR Killeen GF Nkwengulila G Knols BGJ Ferguson HM Nature beats nurture: a cast study of the physiological fitness of free-living and laboratory-reared male *Anopheles gambiae *s.l J Exp Biol 2007 210 2939 2947 10.1242/jeb.005033 17690243

[B19] Howard AFV Koenraadt CJM Farenhorst M Knols BGJ Takken W Pyrethroid resistance in *Anopheles gambiae *leads to increased susceptibility to entomopathogenic fungi *Metarhizium anisopliae *and *Beauveria bassiana* Malar J 2010 9 168 10.1186/1475-2875-9-168 20553597 PMC2898789

[B20] Vatandoost H Shamspour S Abai MR Relative efficacy of different synthetic pyrethroids impregnated fabrics (ITNs) against *Anopheles stephensi *in Iran Pak J Biol Sci 2006 9 503 506 10.3923/pjbs.2006.503.506

[B21] Fillinger U Lindsay SW Suppression of exposure to malaria vectors by an order of magnitude using microbial larvicides in rural Kenya Trop Med Int Health 2006 11 1629 1642 10.1111/j.1365-3156.2006.01733.x 17054742

[B22] Karch S Manzambi ZA Salaun JJ Field trials with VectoLex (*Bacillus sphaericus*) and VectoBac (*Bacillus thuringiensis *(H-14)) against *Anopheles gambiae *and *Culex quinquefasciatus *breeding in Zaire J Am Mosqu Control Assoc 1991 7 176 179 1895075

[B23] Itoh T Evaluation of long-lasting insecticidal nets after 2 years household use Trop Med Int Health 2005 10 1321 1326 10.1111/j.1365-3156.2005.01523.x 16359414

[B24] Lwetoijera DW Sumaye RD Madumla EP Kavishe DR Mnyone LL Russell TL Okumu FO An extra-domiciliary method of delivering entomopathogenic fungi, *Metarhizium anisopliae *IP 46 against malaria vectors, *Anopheles arabiensis* Parasit Vectors 2010 3 18 10.1186/1756-3305-3-18 20233423 PMC2848008

[B25] Okumu FO Madumla EP John A Lwetoijera DW Sumaye RD Attracting, trapping and killing disease-transmitting mosquitoes using odour-baited stations - The Ifakara Odour-Baited Stations Parasit Vectors 2010 3 12 10.1186/1756-3305-3-12 20193085 PMC2838860

